# Anticoagulation in chronic thromboembolic pulmonary hypertension: an updated systematic review and meta-analysis

**DOI:** 10.1007/s11739-025-04257-y

**Published:** 2026-01-20

**Authors:** Filippo Catalani, Emanuele Valeriani, Walter Ageno, Elena Campello, Arianna Pannunzio, Pasquale Pignatelli, Ettore Sgro, Sandor Györik

**Affiliations:** 1 Department of Internal Medicine, Regional Hospital of Bellinzona e Valli, Ente Ospedaliero Cantonale (EOC), Bellinzona, Switzerland; 2 Department of General Surgery, Surgical Specialty and Anesthesiology, Sapienza University of Rome, Rome, Italy; 3 Department of Internal Medicine, Endocrino-Metabolic Sciences, and Infectious Disease, Azienda Ospedaliero-Universitaria Policlinico Umberto I, Rome, Italy; 4 Department of Medicine, University of Padua, Padua, Italy; 5 Internal Medicine 1, Department of Medicine, University of Padova, Padua, Italy

**Keywords:** CTEPH, Pulmonary embolism, Anticoagulation, Direct oral anticoagulants, DOAC, VKA

## Abstract

Chronic thromboembolic pulmonary hypertension (CTEPH) is an uncommon complication of acute pulmonary embolism (PE), resulting in elevated pulmonary pressure and higher risk of PE recurrence. Therefore, lifelong anticoagulant therapy is mandatory in patients diagnosed with this condition. Despite anticoagulation with vitamin K antagonists (VKAs) has always represented the standard of care in this setting, the spread of direct oral anticoagulants (DOACs) raised the question of the potential applicability of their use also in CTEPH. We performed a systematic review and meta-analysis of randomized and observational studies focusing on patients with CTEPH treated with either VKAs or DOACs. Key clinical outcomes as venous thromboembolism (VTE) recurrence, bleedings (major bleeding, clinically relevant non-major bleeding, and intracranial hemorrhage), and mortality were evaluated. Overall, 12 studies including 4071 patients were selected in the quantitative analysis, 10 had an observational design. We found no difference between DOACs and VKAs for VTE recurrence (RR 0.99, 95% CI 0.40–2.43), overall bleedings (RR 0.77, 95% CI 0.45–1.32), and all-cause death (RR 0.58, 95% CI 0.30–1.14). These results were consistent for the aforementioned outcomes also in a sensitivity analysis pooling the results of RCTs and prospective studies only (OR 0.75, 95% CI 0.24–2.38; OR 0.59, 95% CI 0.22–1.56; OR 0.69, 95% CI 0.01–74.68; respectively). DOACs appear to be as effective and safe as VKAs for patients with CTEPH, therefore their use may be considered in the absence of clinical contraindications. Larger randomized controlled trials are warranted to further confirm our findings.

**Supplementary Information:**

The online version contains supplementary material available at 10.1007/s11739-025-04257-y.

## Introduction

Chronic thromboembolic pulmonary hypertension (CTEPH), also known as pulmonary hypertension (PH) group IV, is a late complication of acute pulmonary embolism (PE), deriving from incomplete thrombus resolution leading to chronic obstruction of the pulmonary artery tree together with impaired vascular re-modeling, resulting in small-vessel arteriopathy [1–4]. The overall incidence of CTEPH after a PE is considered to be lower than 3%, [2] thus not justifying a routine screening in all PE survivors, according to current guidelines [5, 6]. Lifelong therapeutic anticoagulation is the cornerstone of CTEPH treatment since PE recurrence and incomplete thrombus resolution are the main pathophysiological characteristics of this disease. Vitamin K antagonists (VKAs) have always represented the standard of treatment in this setting, even after direct oral anticoagulants (DOACs) were recommended as the first therapeutic option for the primary treatment and extended secondary prevention of PE [5, 6]. The preference for VKAs in patients who develop CTEPH is based on the lack of sufficient evidence to support DOACs as valid alternatives. [5] Yet, over the last years, the use of DOACs has become increasingly common also for the treatment of patients with CTEPH in daily clinical practice [7, 8].

Some data from observational cohort studies suggested similar bleeding risk, but higher rates of venous thromboembolism (VTE) recurrence in patients treated with DOACs [8, 9]. On the other hand, more recent studies showed a comparable profile in terms of mortality and VTE recurrence among patients treated with VKAs and DOACs [7, 10], with lower bleeding risk for those on DOACs [7].

Therefore, we carried out a systematic review and meta-analysis with the aim to provide a comprehensive overview of the current literature evaluating efficacy and safety outcomes of anticoagulant therapy with VKAs and DOACs in patients with CTEPH.

## Patients and methods

We conducted this systematic review and meta-analysis according to the Preferred Reporting Items for Systematic reviews and Meta-Analysis (PRISMA) guidelines [11]. As a study-level analysis, no ethical approval was needed. The study protocol of this meta-analysis was registered on PROSPERO (CRD420251053635).

### Search strategy

A systematic literature search was carried out in Embase, MEDLINE, and the Cochrane Central Register of Controlled Trials (CENTRAL) from inception to June 17th, 2025, for randomized controlled trials (RCTs), prospective, and retrospective cohort studies. The search string is available online (*Supplementary material*). To complement our search, all references from selected studies were retrieved and manually reviewed. No language restriction was applied.

### Eligibility criteria

Studies were considered eligible if their patient population consisted of adults with diagnosis of CTEPH treated with anticoagulant therapy with either VKAs or DOACs.

Both, cohort studies and RCTs were included in the literature search and meta-analysis. Prospective and retrospective cohort studies and registries were considered eligible. Each study was required to be available in full text and comprise at least 20 patients. Review articles and case reports were excluded, as well as those studies presenting a cross-sectional design.

### Study outcomes

We evaluated the efficacy of the anticoagulant therapy through the recurrence of venous thromboembolic events, consisting in acute PE and/or deep vein thrombosis and vein thrombosis in other sites. In terms of safety, we evaluated major bleeding, clinically relevant non-major bleeding, and intracranial hemorrhage. If a homogeneous definition of bleeding categorization was not provided, definitions of each included study were used. Finally, all-cause death was also evaluated.

### Study selection, data extraction, and quality assessment

Retrieved studies were imported into Rayyan QCRI management software (https://rayyan.qcri.org). After removal of duplicates, two authors worked independently (FC, EV) for the initial screening of titles and abstracts and then perused the full texts to confirm the eligibility of studies at a second stage. A third author (AP) was consulted to resolve any discordance regarding eligibility of studies.

A predefined spreadsheet was created, where two authors (FC, AP) independently extracted data from eligible studies. A pilot test was performed before the formal initiation of data extraction to ensure coherence. Possible disagreements were resolved by consensus or by discussion with a third review author (EV).

We extracted the data regarding first author’s name, year of publication and design for all the eligible studies, as well as population characteristics and outcomes. Two authors (FC, ES) independently evaluated/assessed the quality of the studies, according to the Newcastle–Ottawa Scale (NOS) [12] for cohort studies and the Revised Cochrane risk-of-bias tool for randomized trials (RoB 2) [13] for controlled trials.

### Strategy for data synthesis

Pooled prevalence estimates with their 95% confidence intervals (CIs) were obtained using a random-effects model based on the inverse variance approach, with the DerSimonian–Laird method applied to estimate *τ*^2^. Similarly, pooled odds ratios (ORs) and corresponding 95% CIs were derived through a random-effects model using the Mantel–Haenszel method. The Sidik–Jonkman approach was adopted for *τ*^2^ estimation, and the Hartung–Knapp adjustment was implemented for the random-effects model. When studies included zero cell counts, a continuity correction of 0.5 was applied. Heterogeneity was evaluated according to the following *I*^2^ thresholds: (i) 0–40% suggesting minimal or no heterogeneity, (ii) 30–60% indicating moderate heterogeneity, (iii) 50–90% reflecting substantial heterogeneity, and (iv) 75–100% representing considerable heterogeneity. Nonetheless, the interpretation of *I*^2^ values also considered the effect size direction, magnitude, and strength of evidence for heterogeneity. If sufficient data were available, subgroup analysis was planned according to the type of recurrent VTE, severity of bleeding (e.g., major bleeding or clinically relevant non-major bleeding), and cause of death. Publication bias was explored by visual inspection of funnel plots of logit-transformed proportions against their standard errors, with funnel plot asymmetry evaluated using Egger’s test. Statistical analysis was conducted in RStudio (version 1.2.5001) using the “meta” and “forest” packages.

## Results

### Systematic review of the literature

**Fig. 1:**
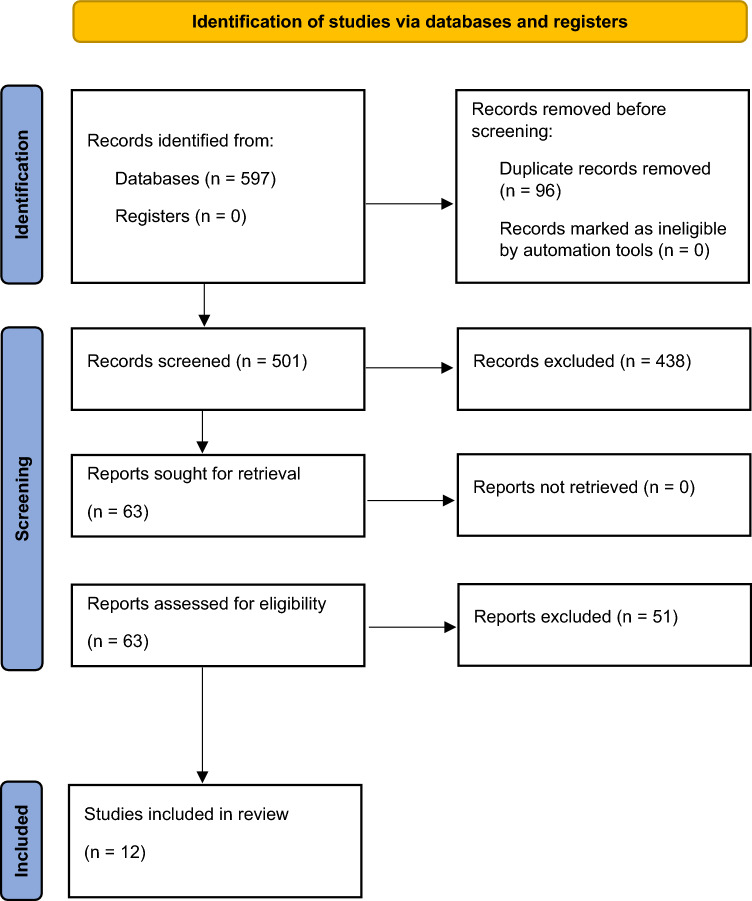
Study selection process according to the search strategy

We identified 597 potential studies and eventually included in the systematic review and quantitative analysis a total of 12 studies. The process of study selection is described in Fig. 1. We classified the included studies into (i) cohort studies (*n* = 10; 3903 patients) (ii) RCTs (*n* = 2; 168 patients), (iii) and prospective cohort studies and RCTs (*n* = 4; 1976 patients). The characteristics of the studies included in the meta-analysis are listed in Table 1. The mean age of patients in individual studies ranged from 45 to 69 years and the prevalence of women ranged from 40% to 85%. The initial mean pulmonary artery pressure (mPAP) ranged from 29 to 48 mmHg. A total of 2777 patients were treated with VKAs and 1294 with DOACs (apixaban = 245, dabigatran = 26, edoxaban = 241, rivaroxaban = 712, unspecified = 70). Other key study characteristics together with the definition of treatment exposures and outcomes used in the individual studies are summarized in the *Supplementary material* (*Table S1* and *Table S2,* respectively).

**Table 1:** Characteristics of the studies included after systematic review of the literature

Author, year	*N*	Study treatment	Study population	Study design	Women (%)	Age (years)
Humbert (2022) [8]	881	Apixaban (*n* = 23)	CTEPH patients	Prospective cohort study	DOACs: 62.1% VKAs: 59.2%	DOACs: 66.0 ± 14.5 VKAs: 66.5 ± 13.6
		Dabigatran (*n* = 10)				
		Edoxaban (*n* = 1)				
		Rivaroxaban (*n* = 164)				
		VKAs (*n* = 683)				
Hosokawa (2024) [10]	72	Edoxaban (*n* = 36)	CTEPH patients	Randomized controlled trial	62.2%	63.6 ± 12.3
		VKAs (*n* = 36)				
Hosokawa (2023) [7]	927	Apixaban (*n* = 154)	CTEPH patients	Prospective cohort study	DOACs: 72.6% VKAs: 69.1%	67 ± 13
		Dabigatran (*n* = 6)				
		Edoxaban (*n* = 157)				
		Rivaroxaban (*n* = 164)				
		VKAs (*n* = 446)				
Barati (2023) [23]	96	Rivaroxaban (*n* = 35)	CTEPH patients who underwent endarterectomy	Randomized controlled trial	DOACs: 40% VKAs: 47.6%	DOACs: 43.2 ± 12.9 VKAs: 47.2 ± 13.2
		VKAs (*n* = 61)				
Takano (2024) [24]	157	Apixaban (*n* = 18)	CTEPH patients	Retrospective cohort study	80%	65 ± 12
		Edoxaban (*n* = 31)				
		Rivaroxaban (*n* = 22)				
		VKAs (*n* = 86)				
Henkens (2013) [25]	60	VKA (*n* = 60)	PH patients	Retrospective cohort study	65%	61 ± 14
Nakano (2024) [26]	50	Apixaban (*n* = 14)	CTEPH patients	Retrospective cohort study	48%	63 ± 14
		Edoxaban (*n* = 10)				
		Rivaroxaban (*n* = 26)				
Sena (2020) [27]	546	Rivaroxaban (*n* = 134)	CTEPH patients	Retrospective cohort study	50.7%	53.5 ± 15
		VKAs (*n* = 412)				
Bunclark (2019) [9]	1000	Apixaban (*n* = 35)	CTEPH patients	Prospective cohort study	DOACs: 47% VKAs: 44%	DOAC: 63 ± 14.6 VKA: 62 ± 15.6
		Dabigatran (*n* = 10)				
		Edoxaban (*n* = 2)				
		Rivaroxaban (*n* = 158)				
		Apixaban or Rivaroxaban (*n* = 1)				
		VKAs (n = 794)				
Jujo-Sanada (2017) [28]	72	VKA (*n* = 72)	CTEPH patients	Retrospective cohort study	84.7%	59.7 ± 11.9
Ikeda (2021) [29]	29	Apixaban (*n* = 1)	CTEPH patients	Retrospective cohort study	DOACs: 78.6% VKAs: 33.3%	DOACs: 69.1 ± 13.7 VKAs: 66.7 ± 13.7
		Edoxaban (*n* = 4)				
		Rivaroxaban (*n* = 9)				
		VKAs (*n* = 15)				
Benzidia (2023) [30]	181	DOAC (*n* = 69)	CTEPH patients	Retrospective cohort study	DOAC: 47% VKA: 62%	DOACs: 65.4 ± 13.5 VKAs: 57.9 ± 17.3
		VKAs (*n* = 112)				

The quality assessment of the included studies is shown in *Table S3* of the *Supplementary material*; the respective tool was selected based on the study design. The risk of bias was low for all included RCTs; among cohort studies, we graded no study as low-quality, four studies as moderate-quality, and six as high-quality studies.

Finally, funnel plots for the study outcomes are showed in *Fig. S1*.

### Study outcomes

#### Recurrent venous thromboembolism

**Fig. 2:**
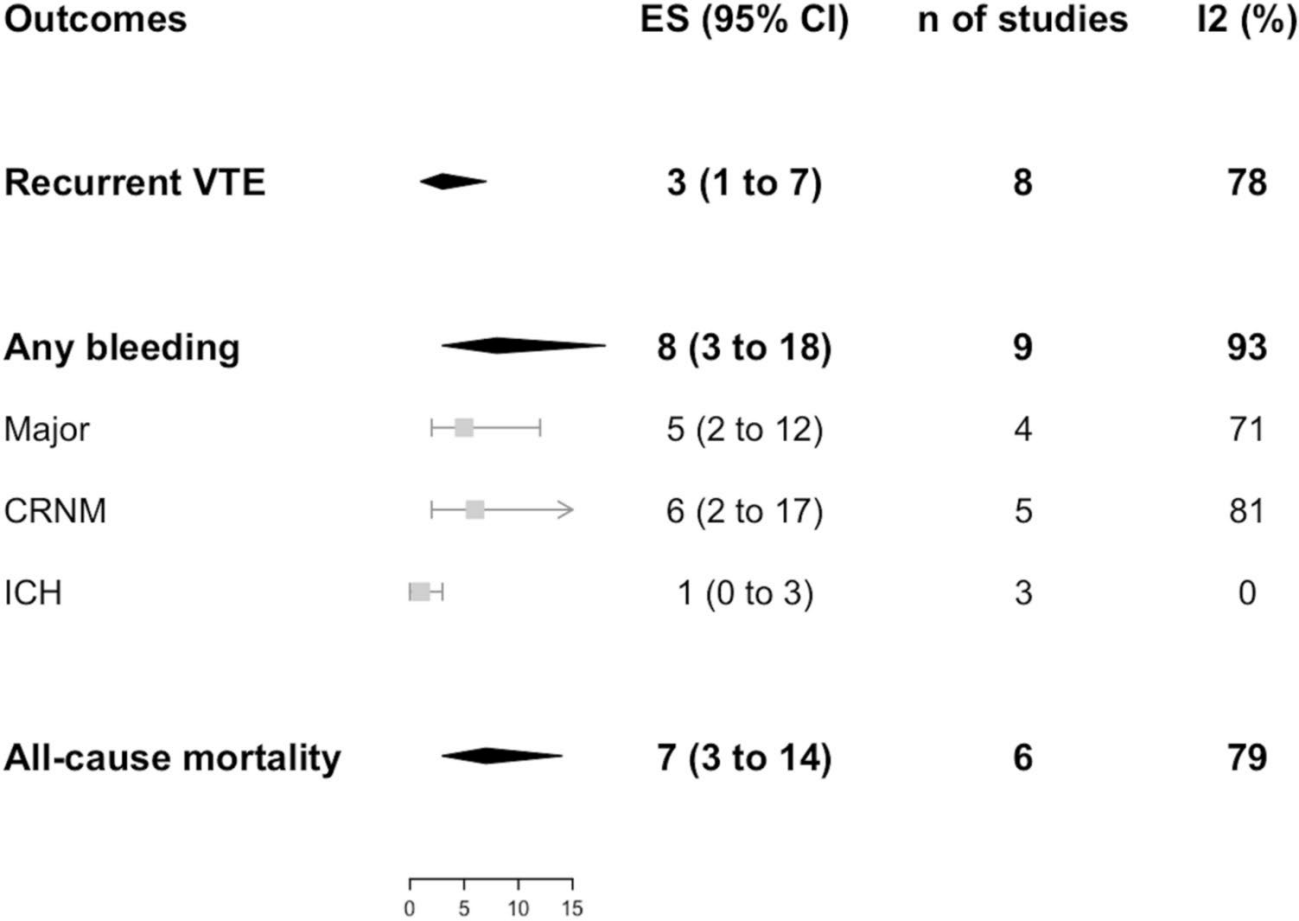
Incidence of outcomes with DOACs

**Fig. 3:**
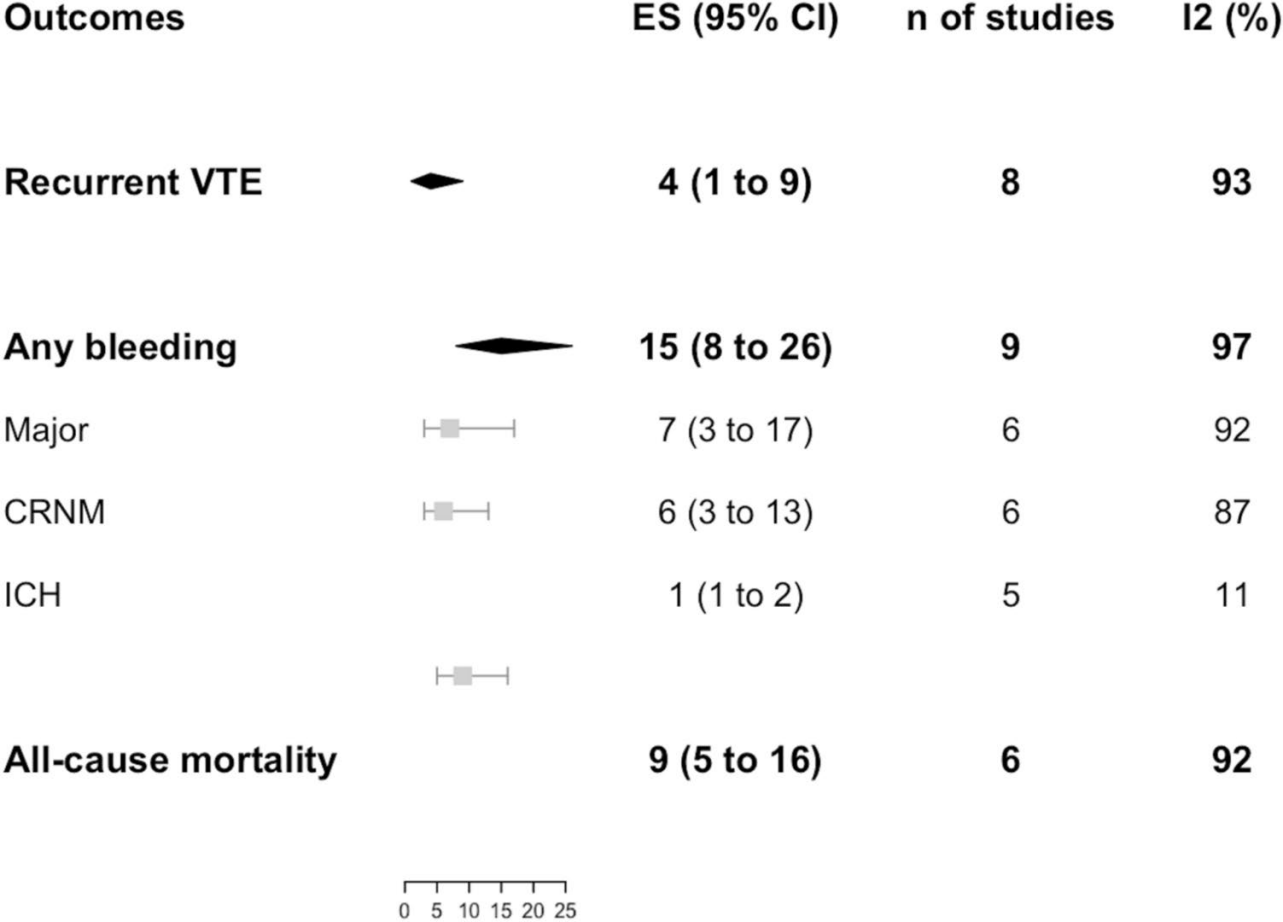
Incidence of the outcomes with VKAs

**Fig. 4:**
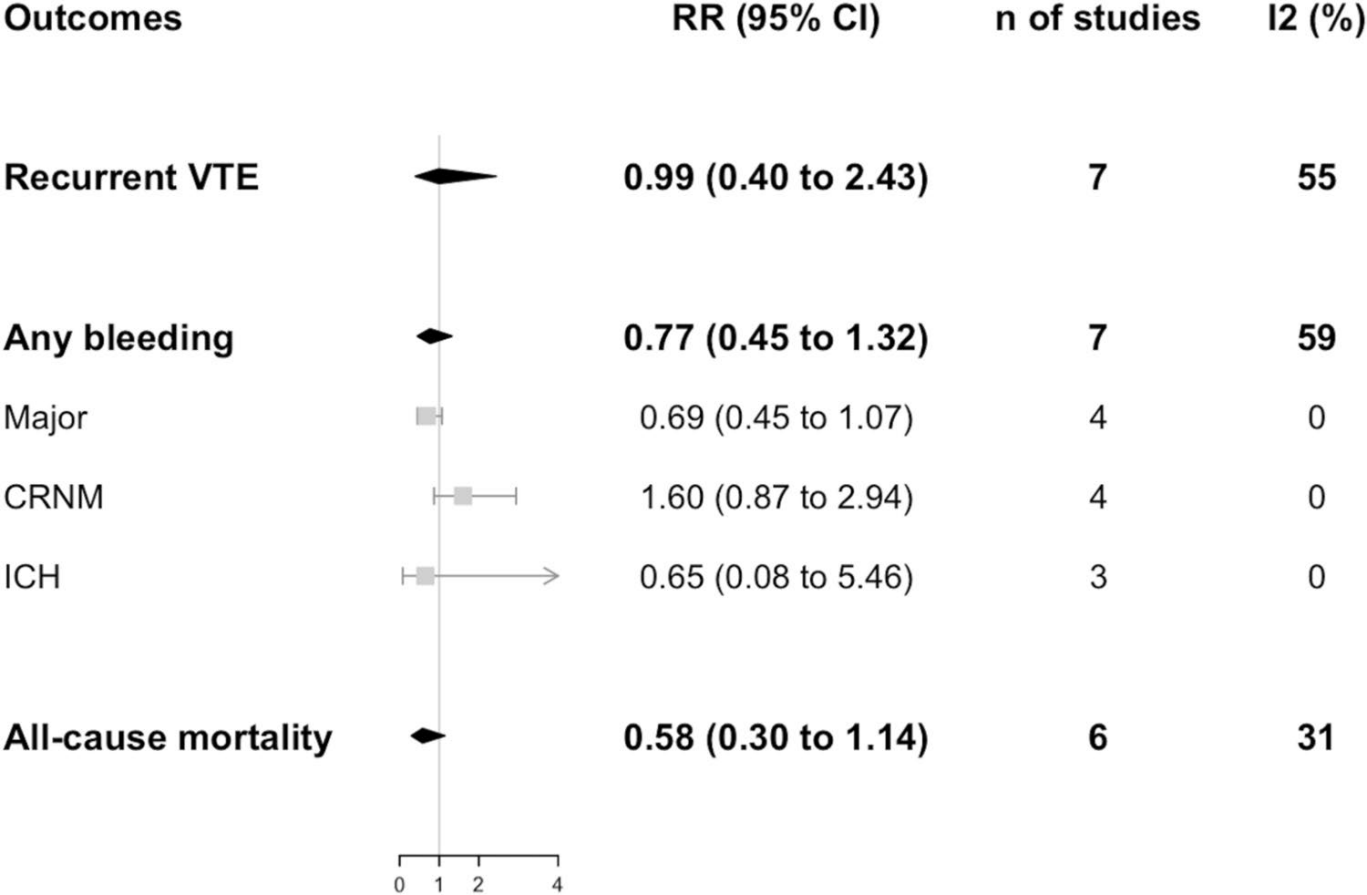
Outcomes DOACs vs. VKAs

Data on recurrent VTE incidence were available from 8 studies for both DOACs (*n* = 1209) and VKAs (*n* = 2616) groups as shown in Figs. 2, 3, respectively. No difference was found between the two anticoagulation strategies (RR 0.99, 95% CI 0.40–2.43; *I*^2^ 55%) as shown in Fig. 4. No difference was observed also in a sensitivity analysis by pooling prospective cohort studies and RCTs only (OR 0.75, 95% CI 0.24–2.38; *I*^2^ 0%, *p* = 0.63) (Fig. S2 a).

#### Bleeding

Data on bleeding incidence were available from 9 studies for both DOACs (*n* = 1223) and VKAs (*n* = 2676) groups, as shown in Figs. 2 and 3, respectively. No difference was found between the two anticoagulation strategies for bleeding overall (RR 0.77, 95% CI 0.45–1.32; *I*^2^ 59%), as well as for the subgroups, namely major and clinically relevant non-major bleedings (RR 0.69, 95% CI 0.45–1.07; *I*^2^ 0% and RR 1.60, 95% CI 0.87–2.94; *I*^2^ 0%, respectively) and intracranial hemorrhage (RR 0.65, 95% CI 0.08–5.46; *I*^2^ 0%) as shown in Fig. 4. Finally, no difference was observed also in a sensitivity analysis by pooling prospective cohort studies and RCTs (OR 0.59, 95% CI 0.22–1.56; *I*^2^ 60%, *p* = 0.06) (Fig. S2 b).

#### All-cause mortality

Data on mortality were available from 6 studies for both DOACs (*n* = 678) and VKAs (*n* = 2098) groups as shown in Figs. 2, 3 respectively. No difference was found between the two anticoagulation strategies (RR 0.58, 95% CI 0.30–1.14; *I*^2^ 31%), as shown in Fig. 4. No difference was observed also in a sensitivity analysis by pooling prospective cohort studies and RCTs only (OR 0.69, 95% CI 0.01–74.68; *I*^2^ 0%, *p* = 0.44) (Fig. S2 c).

## Discussion

The results of this study provide a comprehensive review of the current evidence on anticoagulant treatment of patients with CTEPH, focusing on pivotal clinical outcomes from studies selected on strict inclusion criteria and, to the best of our knowledge, it represents the most updated review on a topic of great clinical interest.

The key results of this work are displayed in the **Central Illustration** and can be summarized as follows: DOACs appear to be as effective and safe as VKAs in the treatment of CTEPH, with no significant difference in mortality and without increased recurrent thromboembolism. The robustness of the results was further supported by sensitivity analysis conducted separately for RCTs and prospective cohort studies, which yielded consistent findings across study designs.

Thanks to their greater ease of use, DOACs are gaining ground over VKAs, despite the lack of clear evidence in their favor. This unmet clinical need has been addressed in the previous meta-analyses; however, these were hampered by the inclusion of a limited number of studies and in some cases only partial results, such as conference abstracts; [14] by restriction to specific study designs (i.e., RCTs and prospective cohort studies), [15] or by the lack of bleeding severity stratification and incorporation of the most recent evidence, [16] thus leading to divergent conclusions. Our systematic review and meta-analysis supports the rationale that the management of CTEPH should focus on treating the underlying disease, namely PE; in this context, except for cases with clear contraindications to their use, DOACs have proven to be as effective and safe as VKAs. Our findings may further reassure clinicians in their clinical practice regarding the use of DOACs as a viable anticoagulant strategy also for the treatment of CTEPH, as is already the case for most current indications, including atrial fibrillation and VTE [6, 17–19].

With respect to safety, this meta-analysis detected a trend toward lower rates of major bleeding for DOACs, showing no significant difference in either overall bleedings or intracranial hemorrhage. This result may not sound unexpected, considering the widely recognized safer profile of DOACs compared to VKAs in terms of bleeding across the approved indications [20–22]. This could be partly explained by the consistently lower proportion of patients treated with DOACs compared to those on VKAs (more than the double) in the included studies: this aspect may have contributed to the lack of statistical significance observed for the aforementioned outcome. Importantly, restriction to prospective studies with balanced patient allocation across the two anticoagulant regimens highlighted a trend toward a superior safety profile for DOACs, [10, 23] with some analyses demonstrating a clear clinical advantage over VKAs [7].

Our study presents several limitations: first, the heterogeneity of the included studies and their design, which is mostly observational and retrospective with relative scarcity of large-scale prospective studies conducted in broad patient populations; second, evidence of a treatment imbalance favoring VKAs over DOACs among the included population, which might have prevented the attainment of significant differences between the study groups and may therefore limit the generalizability of our findings; third, the insufficient reporting in the included studies of potential variations in anticoagulation strategies within study groups, including different INR targets (many centers favor an INR between 2.5 and 3.5) for patients on VKAs and variable dosing regimens for those receiving DOACs, which may have influenced the observed outcomes; fourth, assessing the data only at the study level is an inherent limitation of study-level meta-analysis as it prevents more detailed investigations into how individual patient characteristics (such as specific comorbidities) may influence the outcomes. On the other hand, our systematic review and meta-analysis based on a rigorous methodology and strict inclusion criteria provides the most recent and complete evidence on a topic of current interest, covering a field with limited knowledge.

In conclusion, according to our data, the use of DOACs for the treatment of CTEPH appears to be equally effective and safe compared to VKAs; however, given the limitations of the study design, these findings should be interpreted with caution. Finally, even if the optimal anticoagulation strategy for CTEPH patients has not been proved, clinicians may be reasonably reassured when prescribing DOACs in this clinical setting, at least in the absence of concomitant contraindications, such as the antiphospholipid syndrome or the presence of mechanical heart valves. Further data from well-designed, large, multi-center clinical trials are strongly needed to provide conclusive evidence and to determine whether DOACs will be suitable to become the standard of care in CTEPH.

## Supplementary Information

Below is the link to the electronic supplementary material.

Supplementary file1 (DOCX 359 KB)

Supplementary file2

## Data Availability

All data generated or analysed during this study are included in this published article [and its supplementary information files].
