# Correction to: Anticoagulation in chronic thromboembolic pulmonary hypertension: an updated systematic review and meta-analysis

**DOI:** 10.1007/s11739-026-04317-x

**Published:** 2026-03-17

**Authors:** Filippo Catalani, Emanuele Valeriani, Walter Ageno, Elena Campello, Arianna Pannunzio, Pasquale Pignatelli, Ettore Sgro, Sandor Györik

**Affiliations:** 1 Department of Internal Medicine, Regional Hospital of Bellinzona e Valli, Ente Ospedaliero Cantonale (EOC), Bellinzona, Switzerland; 2 Department of General Surgery, Surgical Specialty and Anesthesiology, Sapienza University of Rome, Rome, Italy; 3 Department of Internal Medicine, Endocrino-Metabolic Sciences, and Infectious Disease, Azienda Ospedaliero-Universitaria Policlinico Umberto I, Rome, Italy; 4 Department of Medicine, University of Padua, Padua, Italy; 5 Internal Medicine 1, Department of Medicine, University of Padova, Padua, Italy

**Correction: Internal and Emergency Medicine** 10.1007/s11739-025-04257-y

In this article the Figure 1 appears to be incompleted. It has been corrected. The correct and uncorrected Figure 1 as follow.

**Uncorrected Figure** 
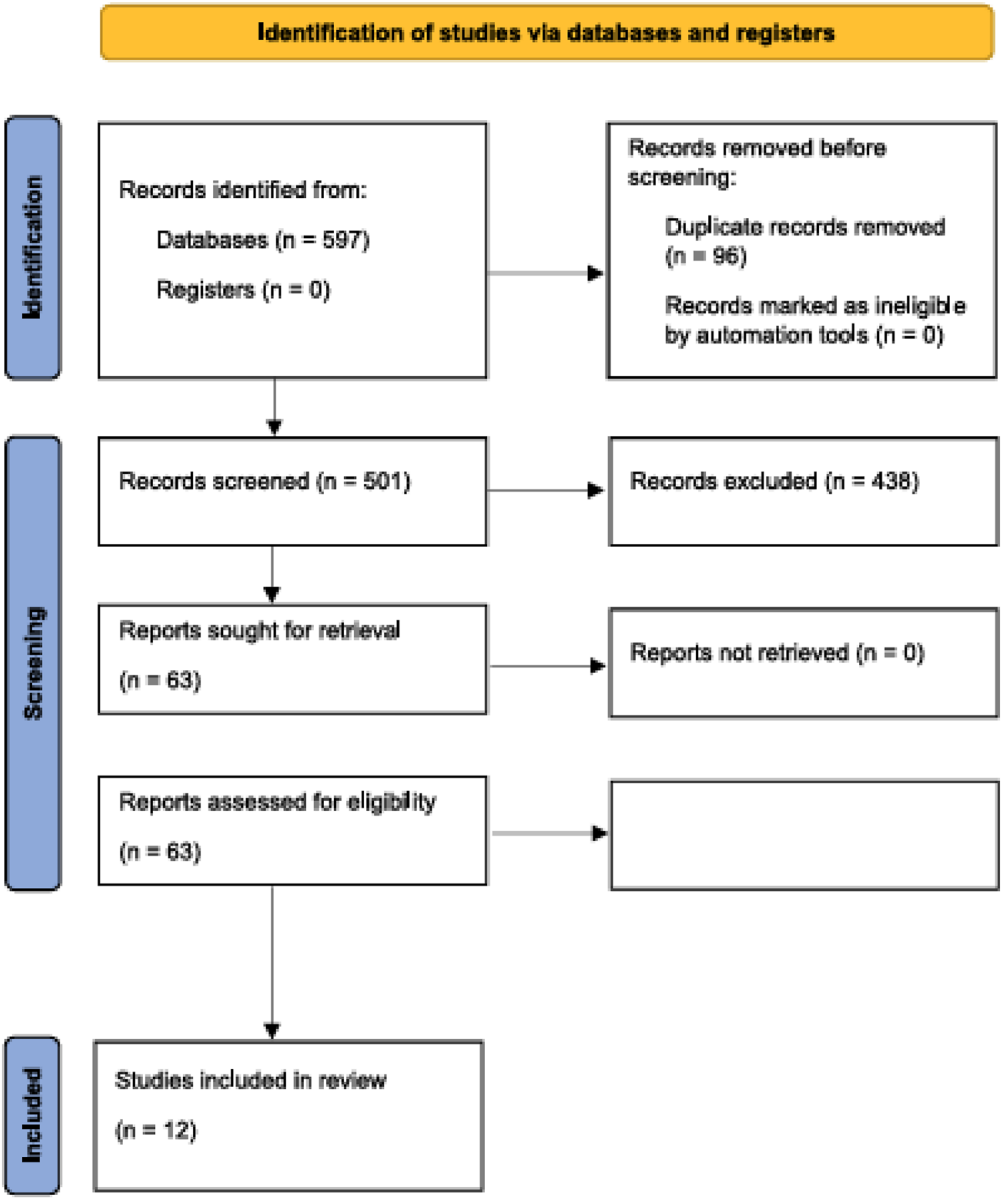


**Fig. 1** Study selection process according to the search strategy

**Corrected Figure** 
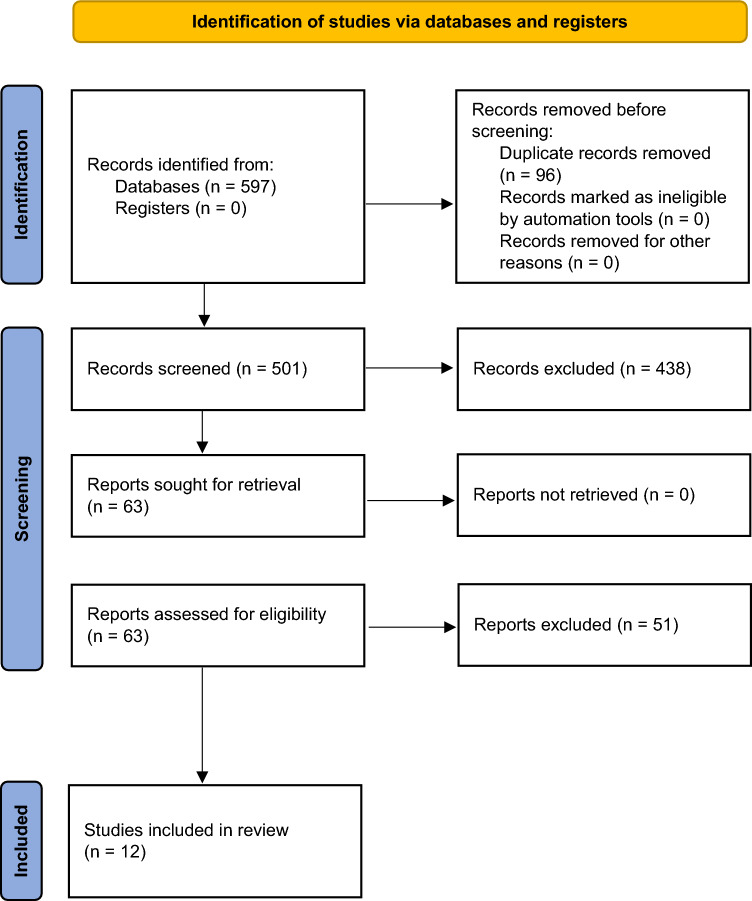


**Fig. 1** Study selection process according to the search strategy

Also, the Supplementary Material and the Central Illustration are not available in the online published version. It has been added.

The original article has been corrected.

