# Supplementary material for: Anticoagulation in chronic thromboembolic pulmonary hypertension: an updated systematic review and meta-analysis
Source: Intern Emerg Med. 2026 Jan 20;21(3):971–8. doi: 10.1007/s11739-025-04257-y (PMC13144224; doi:10.1007/s11739-025-04257-y)
Supplement: Supplementary file 1 — Supplementary file1 (DOCX 359 KB) [file 11739_2025_4257_MOESM1_ESM.docx]

**Supplementary material**

*Fig. S1* Sensitivity analysis

*a*. Recurrent VTE: DOACs vs. VKAs


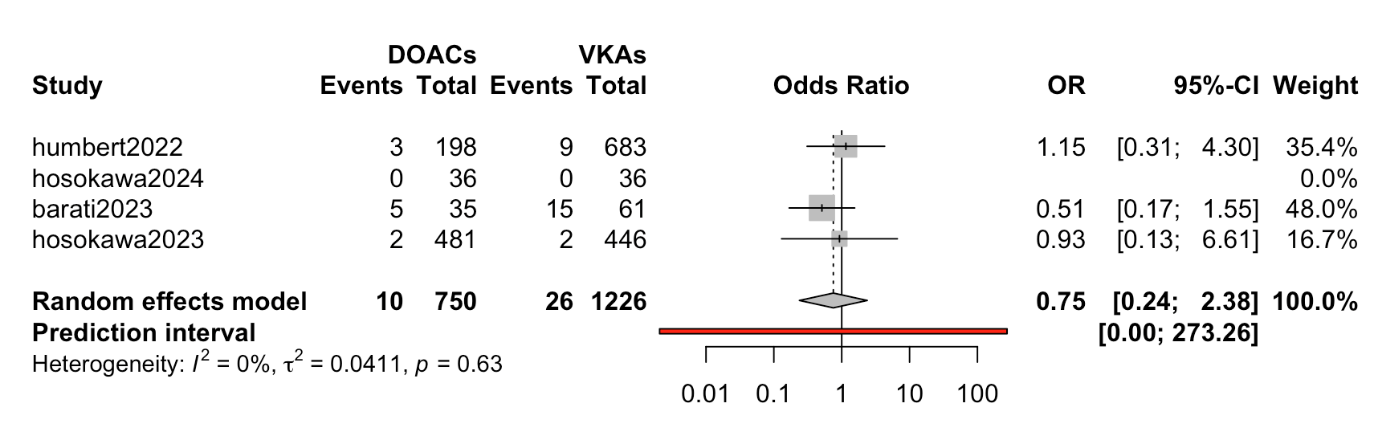


*b*. Any bleeding: DOACs vs. VKAs


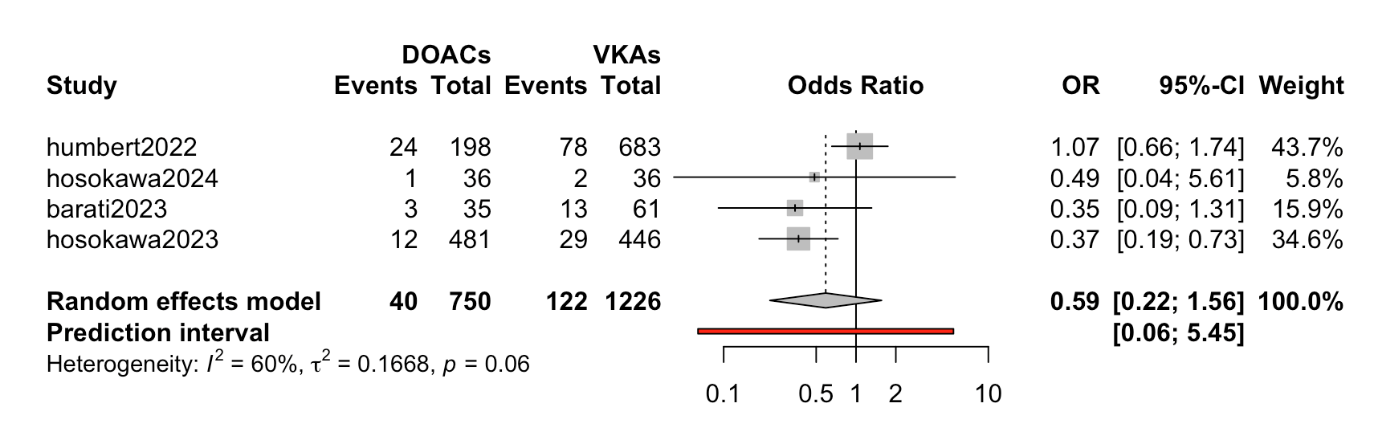


*c*. All-cause mortality: DOACs vs. VKAs


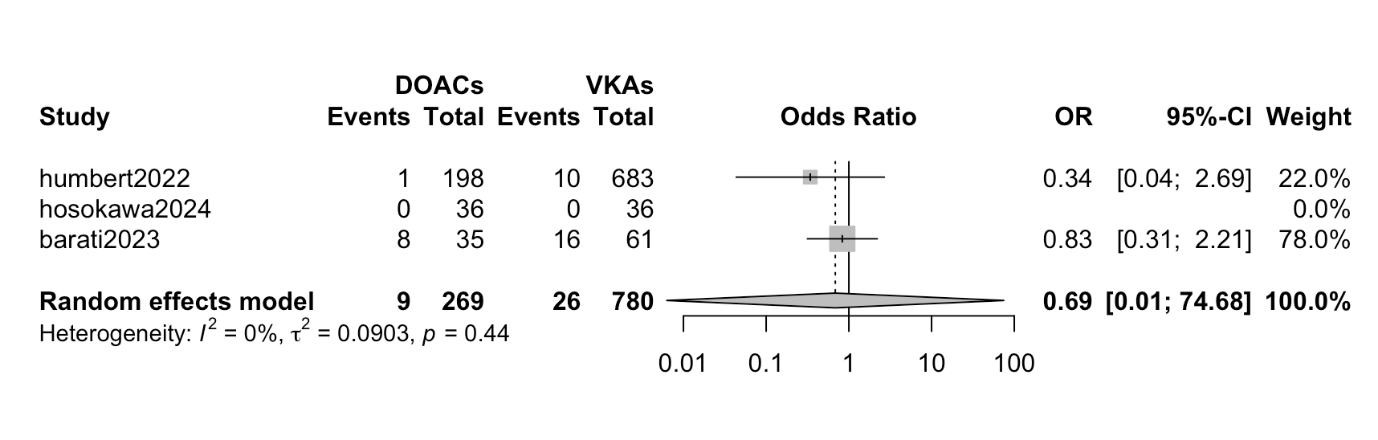


*Table S1.* Countries and years of inclusion

| **Author, year** | **Years of inclusion** | **Country** | **Intervention** | **Collection of data** | **Multicentre** |
| --- | --- | --- | --- | --- | --- |
| Humbert, 2022 | 2014-2018 | France (EXPERT study) | No | Prospective cohort study | Yes |
| Hosokawa, 2024 | 2021-2023 | Japan (KABUKI trial) | Yes | RCT | Yes |
| Hosokawa, 2023 | 2018-2021 | Japan | No | Prospective cohort study | Yes |
| Barati, 2023 | Unspecified | Iran | Yes | RCT | No |
| Takano, 2024 | 2009-2020 | Japan | No | Retrospective cohort study | No |
| Henkens, 2013 | 1997-2009 | Netherlands | No | Retrospective cohort study | No |
| Nakano, 2024 | 2010-2021 | Japan | No | Retrospective cohort study | No |
| Sena, 2020 | 2011-2018 | Turkey | No | Retrospective cohort study | No |
| Bunclark, 2019 | 2007-2018 | UK | No | Prospective cohort study | Yes |
| Jujo-Sanada, 2017 | 2011-2015 | Japan | No | Retrospective cohort study | No |
| Ikeda, 2021 | 2016-2019 | Japan | No | Retrospective cohort study | Yes |
| Benzidia, 2023 | 2001-2021 | Canada | No | Retrospective cohort study | No |

RCT: randomized controlled trial

*Table S2*. Definitions of outcomes and duration of follow-up

| **Author, year** | **Efficacy outcome results** | **Safety outcome results** | **Follow-up** |
| --- | --- | --- | --- |
| Humbert, 2022 | VTE recurrence | Any bleeding  All-cause mortality | VKA: 532 days (median)  DOAC: 465 days (median) |
| Hosokawa, 2024 | VTE recurrence | Major bleeding  Minor bleeding  All-cause mortality | 336 days (median) |
| Hosokawa, 2023 | VTE recurrence | Any bleeding | VKA: 828.7 days (mean)  DOAC: 736.7 days (mean) |
| Barati, 2023 | VTE recurrence | Major bleeding  Minor bleeding  All-cause mortality | 6 months (mean) |
| Takano, 2024 | PH recurrence | - | 45.5 months (mean) |
| Henkens, 2013 | - | Major bleeding  CRNMB  ICH | 1825 days (mean) |
| Nakano, 2024 | VTE recurrence | Major bleeding  CRNMB | 848.8 days (mean) |
| Sena, 2020 | VTE recurrence | Major bleeding  CRNMB  ICH  All-cause mortality | 108 months (mean) |
| Bunclark, 2019 | VTE recurrence | Major bleeding  CRNMB  ICH  All-cause mortality | 612 days (mean) |
| Jujo-Sanada, 2017 | VTE recurrence | Major bleeding  CRNMB  ICH | 43.2 months (mean) |
| Ikeda, 2021 | - | Major bleeding  CRNMB | 180 days (mean) |
| Benzidia, 2023 | VTE recurrence | Major bleeding  CRNMB  ICH  All-cause mortality | VKA: 2153 days (mean)  DOAC: 730 days (mean) |

VTE: venous thromboembolism; VKA: vitamin K antagonist; DOAC: direct oral anticoagulant; CRNMB: clinically relevant non-major bleeding; ICH: intracranial haemorrhage

*Table S3.* The risk of bias assessment of the included studies

| **The Revised Cochrane risk-of-bias tool for randomized controlled trials in the meta-analysis** | | | | | | | | | | | |
| --- | --- | --- | --- | --- | --- | --- | --- | --- | --- | --- | --- |
| **Author, year** | **Bias arising from the randomization process** | **Bias due to deviations from intended interventions** | | **Bias due to missing data** | | **Bias in measurement of outcomes** | | **Bias in selection of the reported result** | | **low/moderate/**  **serious/critical** | |
| Hosokawa, 2024 | Moderate | Moderate | | Low | | Low | | Low | | Low | |
| Barati, 2023 | Moderate | Moderate | | Low | | Low | | Low | | Low | |
| **The Newcastle-Ottawa Scale for assessing the quality of nonrandomized studies in the meta-analysis** | | | | | | | | | | | |
|  | **Selection** | | | | | **Comparability of the cohort** | **Outcome** | | | | **Total score** |
| **Author, year** | **Representativeness of the exposed cohort** | **Selection of the non-exposed cohort** | **Ascertainment of exposure** | | **Outcome not present at baseline** |  | **Assessment of outcome** | | **Enough follow up duration** | **Adequate follow-up** |  |
| Humbert, 2022 | * | * | * | | * | * | * | | * |  | 7 |
| Hosokawa, 2023 | * | * | * | | * | * | * | | * |  | 7 |
| Takano, 2024 | * |  | * | | * | * | * | | * |  | 6 |
| Henkens, 2013 | * |  | * | | * |  | * | | * |  | 5 |
| Nakano, 2024 | * |  | * | | * | * | * | | * |  | 6 |
| Sena, 2020 | * | * | * | | * | * | * | | * | * | 8 |
| Bunclark, 2019 | * | * | * | | * | * | * | | * |  | 7 |
| Jujo-Sanada, 2017 | * |  | * | | * |  | * | | * |  | 5 |
| Ikeda, 2021 | * | * | * | | * | * | * | | * | * | 8 |
| Benzidia, 2023 | * | * | * | | * | * | * | | * |  | 7 |

**Literature search strategy**

1 ((thromb* or embol*) adj3 (chronic or pulmon* or lung or hypertension or type)).mp.

2 warfarin.mp. [mp=ti, ot, ab, fx, sh, hw, kw, tn, dm, mf, dv, kf, dq, bt, nm, ox, px, rx, ui, sy, ux, mx]

3 apixaban.mp. [mp=ti, ot, ab, fx, sh, hw, kw, tn, dm, mf, dv, kf, dq, bt, nm, ox, px, rx, ui, sy, ux, mx]

4 edoxaban.mp. [mp=ti, ot, ab, fx, sh, hw, kw, tn, dm, mf, dv, kf, dq, bt, nm, ox, px, rx, ui, sy, ux, mx]

5 dabigatran.mp. [mp=ti, ot, ab, fx, sh, hw, kw, tn, dm, mf, dv, kf, dq, bt, nm, ox, px, rx, ui, sy, ux, mx]

6 rivaroxaban.mp. [mp=ti, ot, ab, fx, sh, hw, kw, tn, dm, mf, dv, kf, dq, bt, nm, ox, px, rx, ui, sy, ux, mx]

7 acenocoumarol.mp. [mp=ti, ot, ab, fx, sh, hw, kw, tn, dm, mf, dv, kf, dq, bt, nm, ox, px, rx, ui, sy, ux, mx]

8 phenprocoumon.mp. [mp=ti, ot, ab, fx, sh, hw, kw, tn, dm, mf, dv, kf, dq, bt, nm, ox, px, rx, ui, sy, ux, mx]

9 2 or 3 or 4 or 5 or 6 or 7 or 8

10 1 and 9

11 (thromboembol* adj3 (chronic or pulmon* or hypertension or type)).mp.

12 CTEPH.mp. [mp=ti, ot, ab, fx, sh, hw, kw, tn, dm, mf, dv, kf, dq, bt, nm, ox, px, rx, an, ui, sy, ux, mx]

13 11 or 12

14 10 and 13

15 limit 14 to full text
