# Supplementary material for: Anticoagulation in chronic thromboembolic pulmonary hypertension: an updated systematic review and meta-analysis
Source: Intern Emerg Med. 2026 Jan 20;21(3):971–8. doi: 10.1007/s11739-025-04257-y (PMC13144224; doi:10.1007/s11739-025-04257-y)
Supplement: Supplementary file 2 — Supplementary file2 [file 11739_2025_4257_MOESM2_ESM.pptx]

## Slide 1
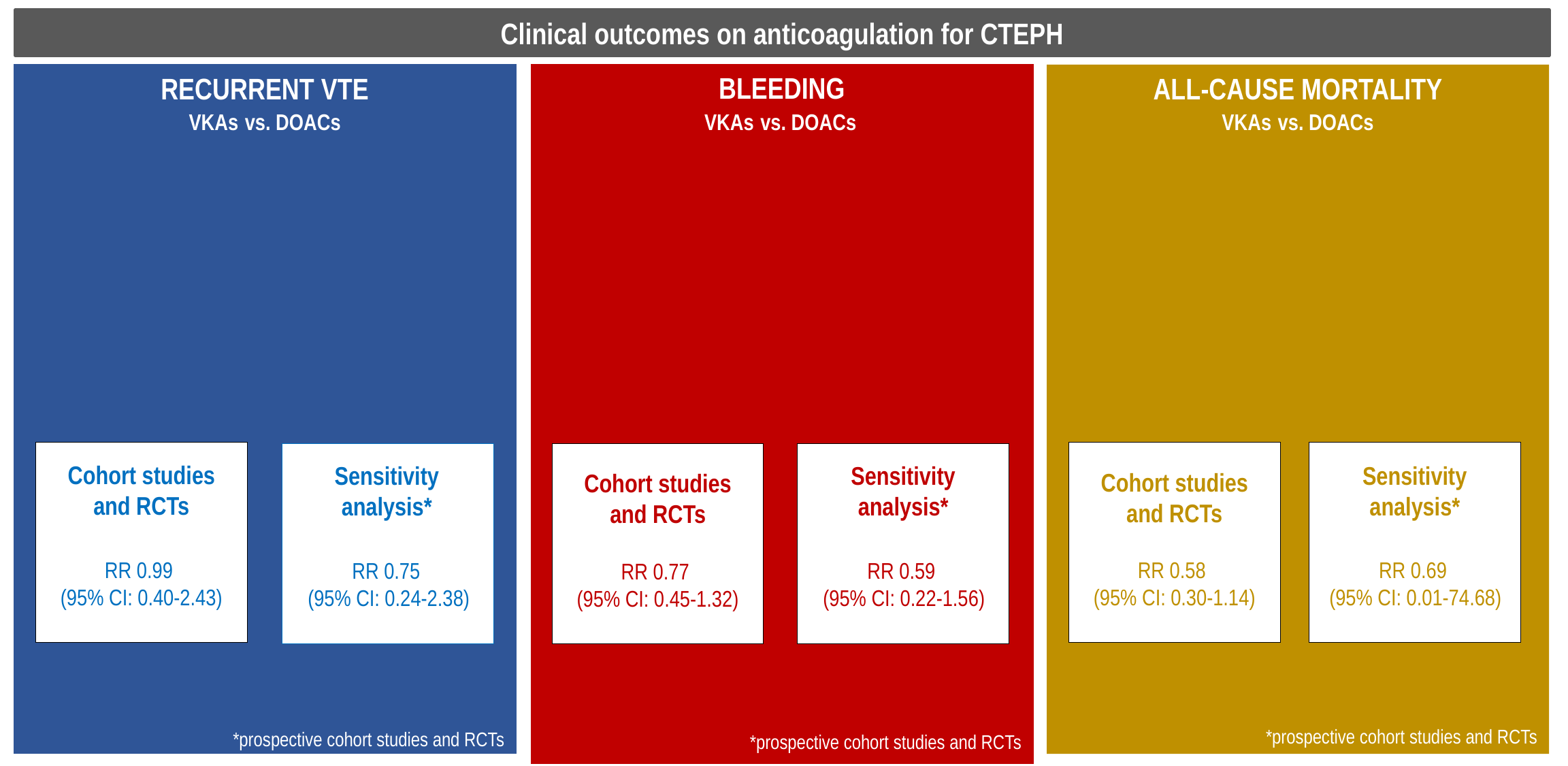

Clinical outcomes on anticoagulation for CTEPH
BLEEDING
ALL-CAUSE MORTALITY
RECURRENT VTE
VKAs vs. DOACs
VKAs vs. DOACs
VKAs vs. DOACs
Cohort studies
and RCTs
Sensitivity analysis*
Sensitivity analysis*
Sensitivity analysis*
Cohort studies
and RCTs
Cohort studies
and RCTs
RR 0.69
(95% CI: 0.01-74.68)
RR 0.99
(95% CI: 0.40-2.43)
RR 0.58
(95% CI: 0.30-1.14)
RR 0.75
(95% CI: 0.24-2.38)
RR 0.59
(95% CI: 0.22-1.56)
RR 0.77
(95% CI: 0.45-1.32)
*prospective cohort studies and RCTs
*prospective cohort studies and RCTs
*prospective cohort studies and RCTs
